# Unraveling Temperature-Dependent Plasma-Catalyzed CO_2_ Hydrogenation

**DOI:** 10.1021/acs.iecr.3c02827

**Published:** 2023-11-04

**Authors:** Yuxuan Zeng, Guoxing Chen, Bowen Liu, Hao Zhang, Xin Tu

**Affiliations:** † Department of Electrical Engineering and Electronics, University of Liverpool, Liverpool L69 3GJ, U.K.; ‡ Fraunhofer Research Institution for Materials Recycling and Resource Strategies IWKS, Brentanostraße 2a, 63755 Alzenau, Germany; § Shenzhen Institute of Advanced Technology, Chinese Academy of Sciences, Shenzhen 518055, China; ∥ Key Laboratory of Clean Energy and Carbon Neutrality of Zhejiang Province, Jiaxing Research Institute, Zhejiang University, Jiaxing 314031, China; ⊥ Zhejiang University Qingshanhu Energy Research Center, 311305 Hangzhou, China

## Abstract

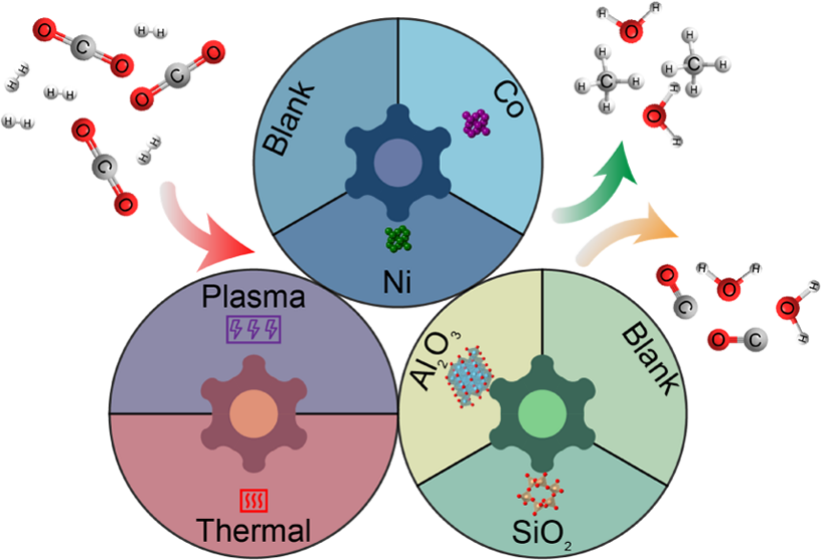

Hydrogenation of carbon dioxide to value-added chemicals and fuels has recently gained increasing attention as a promising route for utilizing carbon dioxide to achieve a sustainable society. In this study, we investigated the hydrogenation of CO_2_ over M/SiO_2_ and M/Al_2_O_3_ (M = Co, Ni) catalysts in a dielectric barrier discharge system at different temperatures. We compared three different reaction modes: plasma alone, thermal catalysis, and plasma catalysis. The coupling of catalysts with plasma demonstrated synergy at different reaction temperatures, surpassing the thermal catalysis and plasma alone modes. The highest CO_2_ conversions under plasma-catalytic conditions at reaction temperatures of 350 and 500 °C were achieved with a Co/SiO_2_ catalyst (66%) and a Ni/Al_2_O_3_ catalyst (68%), respectively. Extensive characterizations were used to analyze the physiochemical characteristics of the catalysts. The results show that plasma power was more efficient than heating power at the same temperature for the CO_2_ hydrogenation. This demonstrates that the performance of CO_2_ hydrogenation can be significantly improved in the presence of plasma at lower temperatures.

## 1 Introduction

The rising concentration of CO_2_ in the atmosphere is a pressing global issue due to its significant and long-term greenhouse effect, which has resulted in catastrophic phenomena, such as climate change, arctic sea ice decline, and ocean acidification. To mitigate greenhouse gas emissions and produce chemicals with a low or even zero carbon footprint, it is critical to directly utilize CO_2_ in conjunction with the CO_2_ capture process. CO_2_ methanation is a highly attractive route for utilizing CO_2_, with the potential to produce key building-block materials for further chemical and fuel synthesis.^1−3^ Various approaches such as electrocatalysis, plasma catalysis, photocatalysis, and thermal catalysis have been explored to convert CO_2_ to methane and CO.^1,4−12^ The most widely accepted mechanism for CO_2_ methanation reaction (R1) is the combination of a reverse water–gas shift (R2) and an exothermic CO methanation (R3).^13,14^
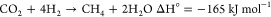
R1
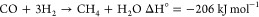
R2
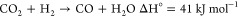
R3

**Figure 1:**
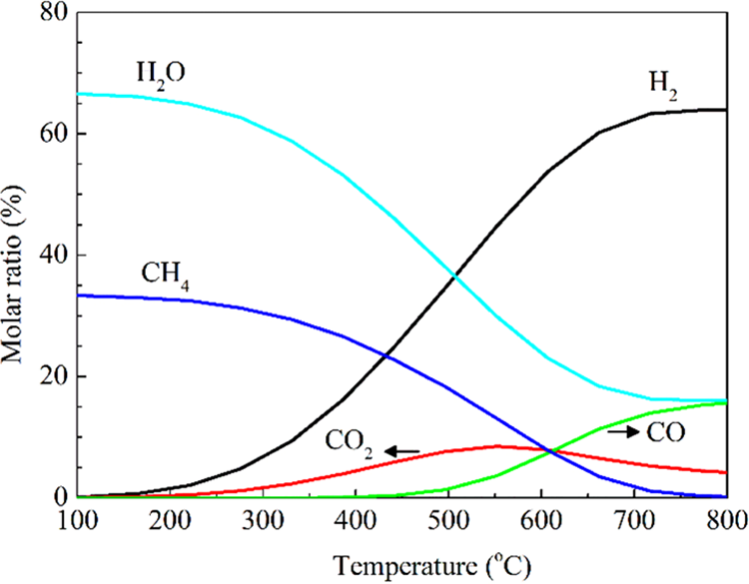
CO_2_ hydrogenation thermodynamic equilibrium composition (H_2_/CO_2_ = 4, 1 bar).

The selectivity for CH_4_ formation decreases with an increasing reaction temperature, as shown in Figure 1. This is because the reverse water–gas shift reaction, which produces CO, becomes more favorable at higher temperatures. In addition, catalyst deactivation caused by coke formation on the catalyst surface is a well-known problem, especially under high-temperature conditions.^15,16^ As a result, hydrogenation of CO_2_ at low temperatures is preferable. Nonthermal plasma (NTP) is a promising way to achieve optimal CO_2_ hydrogenation performance by lowering the reaction temperature while boosting the CH_4_ yield and CO_2_ conversion.

In recent years, there has been growing interest in using NTP technology for chemical and fuel synthesis.^17,18,27,28,19−26^ NTPs are capable of generating highly reactive species and electrons that facilitate thermodynamically unfavorable reactions under mild conditions. When combined with heterogeneous catalysis, NTPs can create a synergistic effect that enhances reaction performance.^29−32^ This combination alters the properties of the catalyst, reduces its activation barrier, and improves the overall energy efficiency and product selectivity of the plasma-catalytic process.^33−35^ Additionally, the fast on/off switching of plasma processes, makes them suitable for integration with irregular and intermittent renewable energy sources, such as solar and wind power.^13,36^ According to the literature, nickel and cobalt-based catalysts have been extensively investigated for CO_2_ methanation due to their low cost and comparable catalytic performance to noble metal-based catalysts.^2,37−42^ These catalysts have been shown to be effective in promoting the CO_2_ methanation reaction at low temperatures and pressures.

Bacariza et al. investigated the performance of Ni-based zeolites (USY zeolites with varying Si/Al ratios) and Ni supported on commercial alumina in an atmospheric pressure dielectric barrier discharge (DBD) reactor.^43^ They found that the CO_2_ conversion and CH_4_ selectivity increased with increasing power input for all tested catalysts. This is consistent with previous research, which has shown that plasma catalysis can enhance the rate of CO_2_ methanation. Zeng et al. examined nickel–alumina catalysts in various reaction conditions and discovered that Ni-based catalysts resulted in higher CO_2_ conversion and CH_4_ yield than Cu and Mn catalysts.^44^ They also found that the addition of Ar to the feed gases can significantly improve the performance of the plasma-catalytic process. Parastaev et al. compared the performance of CeZrO_4_-supported Co and Cu catalysts in CO_2_ hydrogenation using a DBD reactor.^45^ They discovered that the Co-based catalyst was significantly more active and selective for CH_4_ than the Cu-based catalyst. The increased number of H_2_ adsorption sites may have contributed to its increased activity as the Co content rose. Interestingly, the authors confirmed that CO_2_ methanation under plasma conditions primarily involves CO hydrogenation on Co particles and is unaffected by the support. The study of CO_2_ methanation in DBD plasmas has revealed that the overall effectiveness of the process is significantly influenced not only by the catalyst used but also by the operating conditions. Thus, a direct and comprehensive comparison of the various catalysts and their impacts on plasma processes is required. Despite recent advances in plasma catalysis, there is still limited information about the rational design of effective hydrogenation catalysts for this process. Additionally, there has been relatively little research on how reaction temperature affects plasma-catalytic hydrogenation of CO_2_. This is an important area of research that could lead to the development of more efficient and cost-effective CO_2_ methanation processes.

In this study, we designed and developed a temperature controlled DBD reactor, enabling us to investigate chemical reactions at different temperatures and across different reaction modes: plasma alone, thermal catalysis, and plasma catalysis. We investigated plasma-catalytic CO_2_ hydrogenation over the M/SiO_2_ and M/Al_2_O_3_ (M = Co, Ni) catalysts. We also conducted the same reaction using thermal catalysis for comparison. The influence of reaction temperature on the performance of supported Ni- and Co-based catalysts in plasma-catalytic CO_2_ hydrogenation was evaluated in terms of CO_2_ conversion, yields, and selectivity of gaseous products to get new insights into the temperature on plasma-catalyzed CO_2_ hydrogenation.

## 2 Experimental Section

### 2.1 Experimental System

**Figure 2:**
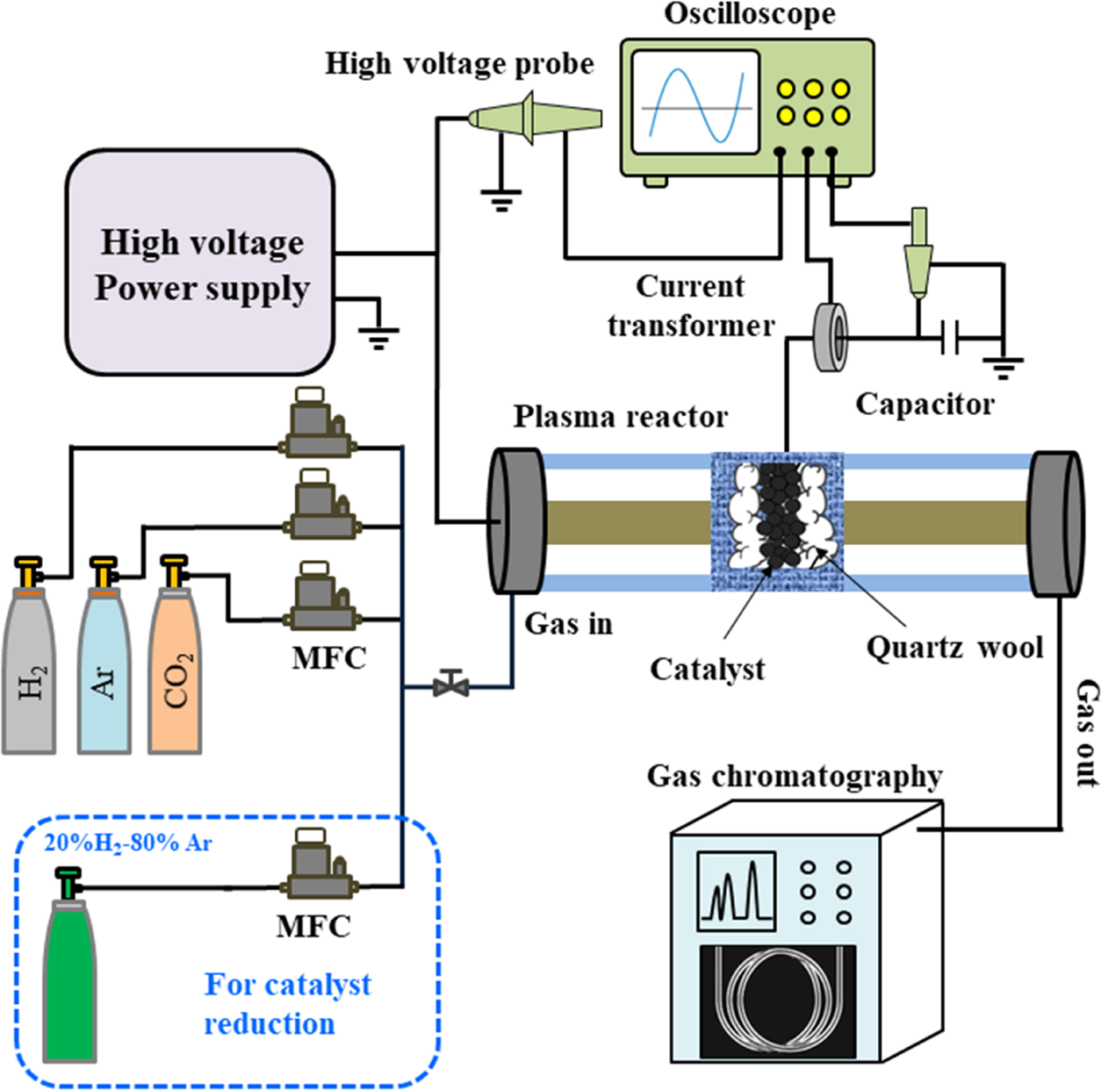
Schematic representation of the experimental setup.

Figure 2 shows the plasma reactor system used for the plasma CO_2_ hydrogenation experiments. More details on the experimental setup can be found in our previous studies.^46^ The DBD reactor was powered by an AC high voltage power supply with a voltage output of 10 kV. The discharge power was determined using the Lissajous figure method and kept constant at 39 W using a homemade real-time plasma power control system. The discharge gap was fixed at 1.5 mm. The feed gas was a mixture of Ar, H_2_, and CO_2_ gases with a mole ratio of 5:4:1, and the total flow rate was fixed at 69.2 mL min^–1^. All gases used were 99.999% pure. The DBD reactor was positioned inside a furnace to allow for the exploration of CO_2_ hydrogenation in three different modes at the same reaction temperature: plasma alone, thermal catalysis, and plasma catalysis.

We prepared 30 wt % M/Al_2_O_3_ (M = Co, Ni) and 30 wt % M/SiO_2_ catalysts using an impregnation approach. Al_2_O_3_ or SiO_2_ powders were used as catalyst supports, and nitrate salts (Alfa Aesar) were used as metal sources. The DBD reactor was filled with quartz wool and 0.4 g of the granular catalyst. In this study, only a portion (∼20 vol %) of the plasma discharge was packed with the catalysts. For thermal-catalytic and plasma-catalytic CO_2_ hydrogenation experiments, 0.4 g of the catalyst was packed in the DBD reactor at a gas hourly space velocity (GHSV) of 10380 mL h^−1^ g^−1^. All of the catalysts were reduced in the same DBD reactor using 20 vol % H_2_-80 vol % Ar (50 mL/min, 40 W, 40 min) prior to thermal-catalytic and plasma-catalytic CO_2_ hydrogenation experiments. The CO_2_ hydrogenation reaction reached a steady state after about 1 h of continuous operation. Gas products were analyzed using a gas chromatograph (Shimadzu GC-2014) equipped with dual detectors (thermal conductivity detector and flame ionization detector) was used. Each measurement was repeated three times. It is important to note that CO and CH_4_ were the major products in this work, accounting for more than 99% of the total products. Light alkanes such as C_2_H_6_, C_3_H_8_, and C_4_H_10_ were produced in such small quantities that their gas yields were assumed to be zero. Unsaturated hydrocarbons were not found.

X-ray diffraction (XRD) patterns of the catalysts were recorded using a Rigaku D–Max 2400 diffractometer equipped with a Cu Kα radiation source, operating in the 2θ range from 20 to 80°. To assess the reducibility of the catalysts, we conducted temperature-programmed reduction of H_2_ (H_2_-TPR) measurements using an automated chemisorption system (Quantac Chrome ChemBET 3000). In the TPR analysis, each sample was initially heated to 400 °C in helium at a flow rate of 20 mL min^–1^ for 1 h. Subsequently, the sample was gradually cooled to 150 °C, purged with hydrogen for 30 min, and then reintroduced helium for 1 h. The BET surface areas of the catalysts were determined by N_2_ adsorption at −196 °C by using the Micrometrics ASAP 2020 instrument (USA).

### 2.2 Definition of Parameters

To evaluate the performances of CO_2_ hydrogenation, the conversion (*C*) of CO_2_ was defined by eq 1
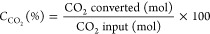
1The selectivity (*S*) and yield (*Y*) of CO and CH_4_ were calculated using eqs 2–5
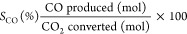
2
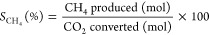
3
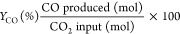
4
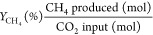
5The carbon balance (*B*_carbon_) and H_2_/CO_2_ molar ratio were defined as follows:
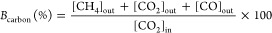
6
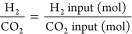
7

## 3 Results and Discussion

### 3.1 XRD Analysis

**Figure 3:**
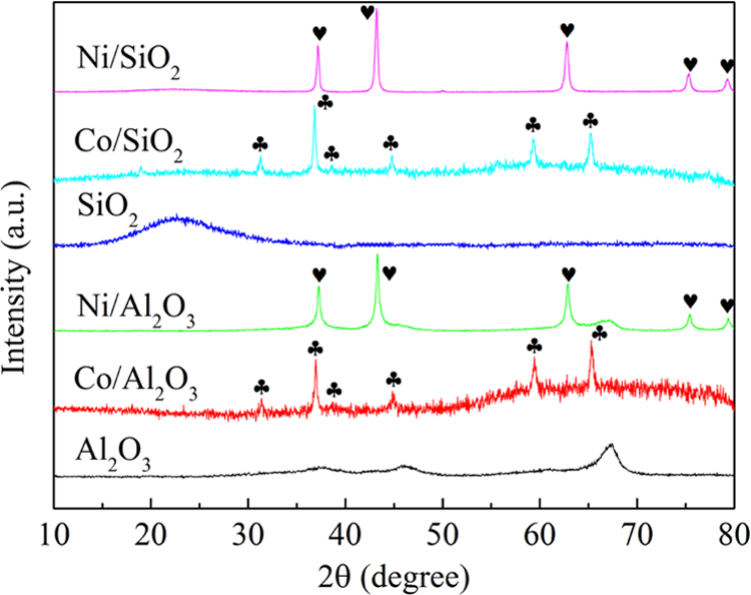
XRD patterns of the fresh catalysts and supports (club symbol Co_3_O_4_, Heart symbol NiO).

Figure 3 presents the XRD patterns of the fresh catalysts and their respective supports. The Al_2_O_3_ support showed three main diffraction peaks at 2θ of 67.0, 45.9, and 37.6°, which are attributed to the cubic alumina crystallite structure (JCPDS 10-425). The SiO_2_ support showed only one broad peak centered at around 22° (JCPDS 29-0085). The Co- and Ni-based catalysts exhibited clear diffraction peaks corresponding to Co_3_O_4_ (JCPDS 42-1467) and NiO (JCPDS 1-75-197), respectively. The average crystallite sizes of the catalysts were determined using the Scherrer equation and are summarized in Table 1. The Ni-based catalysts had smaller crystal sizes than the Co-based samples, and among all of the catalysts investigated, Ni/Al_2_O_3_ showed the smallest average crystal size (29.7 nm).

**Table 1:** Textural Properties of the M/SiO_2_ and M/Al_2_O_3_ (M = Co, Ni) Catalysts and Supports

catalyst	*S*_BET_ (m^2^ g^–1^)	total pore volume (cm^3^ g^–1^)	average crystallite size (nm)
Ni/SiO_2_	133.4	0.94	30.7
Co/SiO_2_	128.5	0.85	45.1
SiO_2_	203.8	1.02	
Ni/Al_2_O_3_	105.5	0.28	29.7
Co/Al_2_O_3_	97.6	0.23	37.5
Al_2_O_3_	174.6	0.44	

### 3.2 N_2_ Adsorption–Desorption Analysis

The nitrogen adsorption–desorption isotherm measurements were used to characterize the texture of the catalysts and supports. The pore-size distribution and specific surface area of the studied supports and catalysts were determined by using the Barrett–Joyner–Halenda (BJH) and Brunauer–Emmett–Teller (BET) methods, respectively. The pore volume and specific surface area of the catalysts were smaller than those of the corresponding supports (Table 1), which can be explained by the crystallite particles of active metal species covering the pores and tunnels in the support. In addition, Ni-based catalysts displayed higher adsorption values than Co-based catalysts, and silica-supported catalysts had larger BET surfaces and pore volumes than alumina-supported catalysts.

### 3.3 Temperature-Programmed Reduction

**Figure 4:**
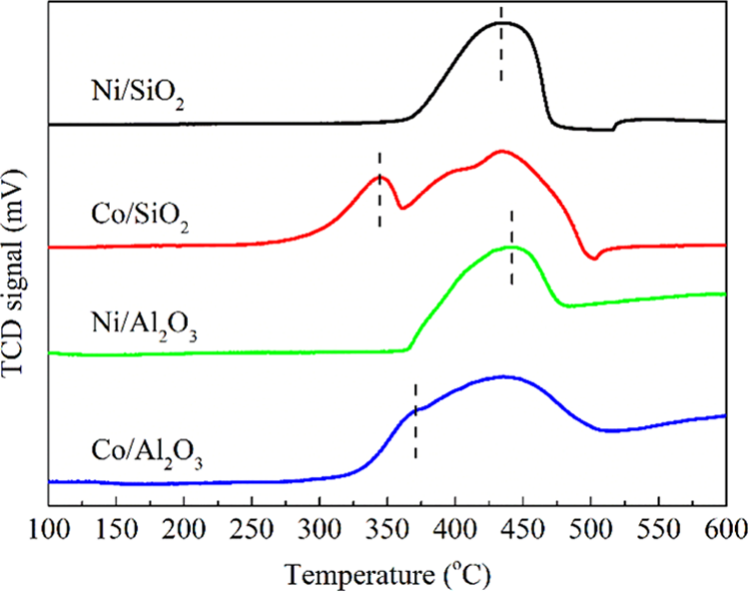
TPR profiles of the M/SiO_2_ and M/Al_2_O_3_ (M = Co, Ni) catalysts.

The reducibility of the catalysts was determined by using H_2_-TPR experiments, and the results are shown in Figure 4. The Ni/SiO_2_ catalyst showed a single reduction peak centered at 430 °C, corresponding to the reduction of NiO particles. The well-defined reduction peak of the Ni/SiO_2_ catalyst suggests that all of the reducible Ni oxides reacted near 430 °C. In comparison, the Co/SiO_2_ catalyst showed a typical feature of Co oxides supported on amorphous SiO_2_. The peaks located at 340 and 430 °C correspond to the reduction of Co_3_O_4_ to Co^2+^ species and Co^2+^ to metallic Co, respectively. It should be noted that the reduction of Co^2+^ was reflected by several superimposed peaks, indicating the different interaction strengths between Co^2+^ species and the SiO_2_ support. The TPR profile of Ni/Al_2_O_3_ showed a peak beginning at 370 °C, corresponding to the reduction of bulk NiO, and other peaks above 500 °C, attributed to the reduction of NiO_*x*_ species or inert Ni-spinel aluminates that react significantly with the alumina support.^47,48^ Since this temperature was higher than the calcination or reaction temperature in this study, it was not taken into consideration. By contrast, the Co/Al_2_O_3_ catalyst exhibited two overlapped peaks at 370 and 430 °C, corresponding to the reduction of Co_3_O_4_ to Co^2+^ species and Co^2+^ to Co^0^, respectively. The TPR results indicated that Ni and Co species interacted more strongly with the Al_2_O_3_ support than SiO_2_, resulting in a broader reduction pattern in the TPR results, particularly at high temperatures. When the active metal species Ni and Co are compared, the reduction of Co_3_O_4_ occurred at a lower temperature than that of NiO. Furthermore, the transformation of Co_3_O_4_ to Co occurred over a broad temperature range, resulting in a wider reduction peak for Co-based catalysts. Overall, the Co-based catalysts (Co/SiO_2_ and Co/Al_2_O_3_) reduced at lower temperatures than the Ni-based ones (Ni/SiO_2_ and Ni/Al_2_O_3_), suggesting a weak interaction between Co species and supports. Similarly, the SiO_2_-supported catalysts (Ni/SiO_2_ and Co/SiO_2_) reduced at slightly lower temperatures than the Al_2_O_3_-supported catalysts (Ni/Al_2_O_3_ and Co/Al_2_O_3_). Based on the TPR results, the catalyst reducibility was in the following order: Co/Al_2_O_3_ < Ni/Al_2_O_3_ < Ni/SiO_2_ < Co/SiO_2_.

### 3.4 CO_2_ Hydrogenation over Different Catalysts

**Figure 5:**
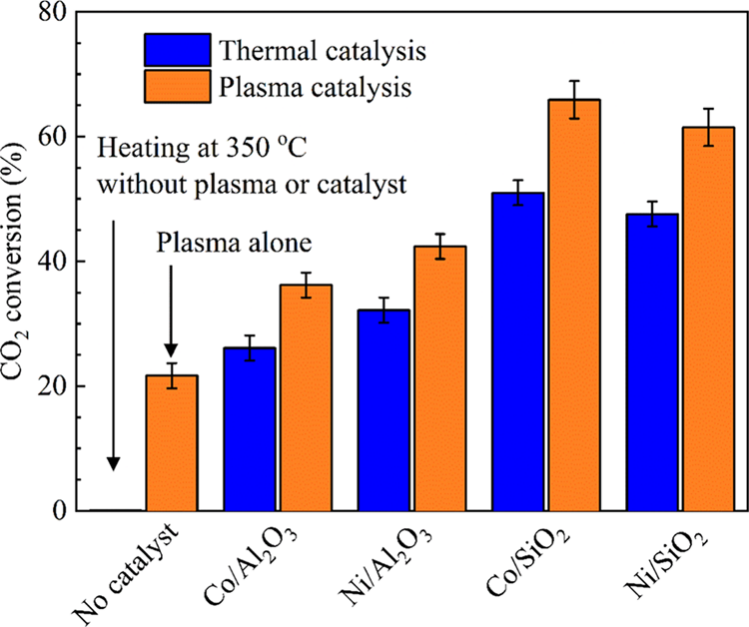
CO_2_ conversion over different catalysts (Ar content: 50%, total flow rate: 69.2 mL min^–1^, H_2_/CO_2_ = 4, reaction temperature: 350 °C).

Figure 5 shows the CO_2_ conversions of the hydrogenation processes over the M/SiO_2_ and M/Al_2_O_3_ (M = Co, Ni) catalysts at a reaction temperature of 350 °C. The addition of catalysts improved the CO_2_ conversion in both thermal- and plasma-catalytic processes, with the CO_2_ conversions across all catalysts in plasma-catalytic processes being higher than those in thermal-catalytic processes. This suggests that plasma–catalyst coupling is beneficial for CO_2_ hydrogenation. One possible explanation for this is that CO_2_ is excited by the DBD plasma before adsorption onto the catalyst surface in the plasma-catalytic process. This lowers the energy barrier for conversion to intermediates compared to thermal catalysis, where an elevated temperature is required to activate the adsorbed surface CO_2_ molecules. Additionally, the catalysts with the SiO_2_ support (Ni/SiO_2_ and Co/SiO_2_) exhibited significantly better CO_2_ conversion than those with the Al_2_O_3_ support (Ni/Al_2_O_3_ and Co/Al_2_O_3_), which may be linked to the larger BET surface area of the catalysts with the SiO_2_ support. Specifically, the CO_2_ conversion of Ni/SiO_2_ was 61.5% in plasma catalysis, which was significantly higher than the CO_2_ conversion of Ni/SiO2 in thermal catalysis (47.4%). The CO_2_ conversion of Co/SiO_2_ was also higher in plasma catalysis (65.8%) than in thermal catalysis (51.0%). In contrast, the CO_2_ conversion of Ni/Al_2_O_3_ and Co/Al_2_O_3_ was similar in plasma catalysis (42.4 and 36.2%) and thermal catalysis (32.1 and 26.0%). These results suggest that the plasma–catalyst coupling is more effective for catalysts with the SiO_2_ support than for the catalysts with the Al_2_O_3_ support. As shown in Table 1, the BET surface areas of Ni/SiO_2_ and Co/SiO_2_ are 133.4 and 128.5 m^2^ g^–1^, respectively, which is significantly higher than the BET surface area of Ni/Al_2_O_3_ (105.5 m^2^ g^–1^) and Co/Al_2_O_3_ (97.6 m^2^ g^–1^). The larger surface area of the catalysts with the SiO_2_ support provides more active sites for the CO_2_ conversion reaction.

**Figure 6:**
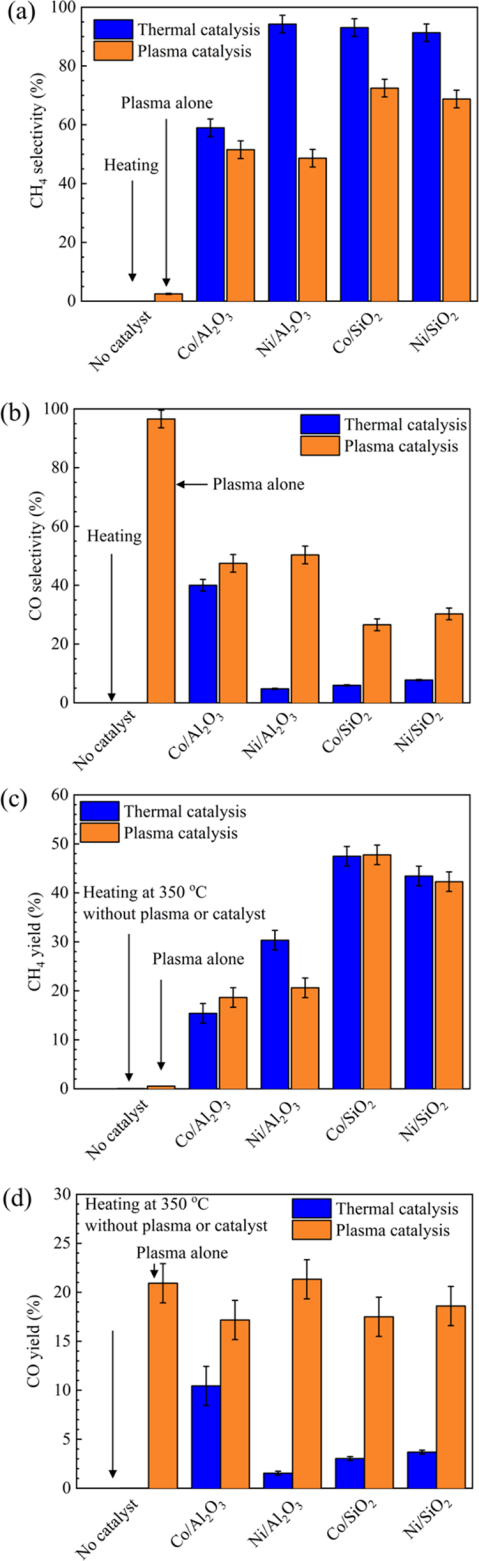
Selectivities (a,b) and yields (c,d) of CH_4_ and CO using different catalysts (Ar content: 50%, H_2_/CO_2_ = 4, reaction temperature = 350 °C, total flow rate = 69.2 mL min^–1^).

Figure 6 summarizes the yields and selectivity of CH_4_ and CO during plasma- and thermal-catalytic CO_2_ hydrogenation processes at a reaction temperature of 350 °C. Remarkably, all the catalysts except Co/Al_2_O_3_ demonstrated high CH_4_ selectivity (>90%) during thermal-catalytic processes, although the Co/Al_2_O_3_ catalyst significantly enhanced the CH_4_ selectivity from 0 to 59% under the same conditions compared to the case without using a catalyst. In comparison to thermal catalysis, the CH_4_ selectivity of plasma-catalytic hydrogenation processes decreased across all catalysts. For example, the CH_4_ selectivity using Co/Al_2_O_3_ slightly dropped from 59 to 52%, as shown in Figure 6. The largest drop occurred over Ni/Al_2_O_3_, where the CH_4_ selectivity decreased from 94.3 to 48.6%. In contrast, the highest CO selectivity (notably 96.6%) was obtained in the plasma alone process. Among the studied catalysts, the Co/Al_2_O_3_ catalyst had the best CO selectivity in the thermal-catalytic mode, while the Ni/Al_2_O_3_ catalyst demonstrated the highest CO selectivity in the plasma-catalytic process. In comparison to the results obtained from thermal catalysis, the CO selectivity over all of the catalysts increased significantly during plasma catalysis. For instance, using Ni/Al_2_O_3_ and Ni/SiO_2_, the CO selectivity was increased by a factor of 957 and 294%, respectively. These findings indicate that the presence of plasma generated a cascade of reactive species, which altered the reaction pathway. The DBD plasma activated the CO_2_ molecules, primarily increasing the level of CO production. Furthermore, the plasma may activate and convert the produced CH_4_ through CH_4_ decomposition or dry reforming of methane (DRM),^49^ reducing overall selectivity toward CH_4_.

As noted in Figure 6c, SiO_2_-supported catalysts exhibited a higher yield of CH_4_ than did Al_2_O_3_-supported catalysts. In both thermal catalysis and plasma catalysis modes, the CH_4_ yields over Co/SiO_2_ and Ni/SiO_2_ were very similar. However, there was no significant difference in plasma-catalytic processes with and without a catalyst (the plasma alone case), as shown in Figure 6d. The CO yields increased in the order: Co/Al_2_O_3_ < Co/SiO_2_ < Ni/SiO_2_ < plasma alone < Ni/Al_2_O_3_. Furthermore, plasma-catalytic hydrogenation yielded more CO than the thermal-catalytic process across all catalysts. The high CO yield using plasma catalysis further confirms that the reactive species present in plasma primarily activated CO_2_ molecules to produce CO, which is in good agreement with the high CO selectivity shown in Figure 6b.

Table 2 shows that the carbon balance is very close to 100% for both thermal- and plasma-catalytic CO_2_ hydrogenation using Co- and Ni-based catalysts at 350 °C. This indicates that the overall selectivity toward CO and CH_4_ is very high at this temperature.

**Table 2:** Carbon Balance of Thermal- and Plasma-Catalytic CO_2_ Hydrogenation Processes over Different Catalysts (Ar Content = 50%, H_2_/CO_2_ = 4, Reaction Temperature: 350 °C, Total Flow Rate = 69.2 mL min^–1^)

	carbon balance (%)	
catalyst	thermal catalysis	plasma catalysis
none		99.8
Co/Al_2_O_3_	99.8	99.7
Ni/Al_2_O_3_	99.7	99.6
Co/SiO_2_	99.5	100
Ni/SiO_2_	99.6	100

**Figure 7:**
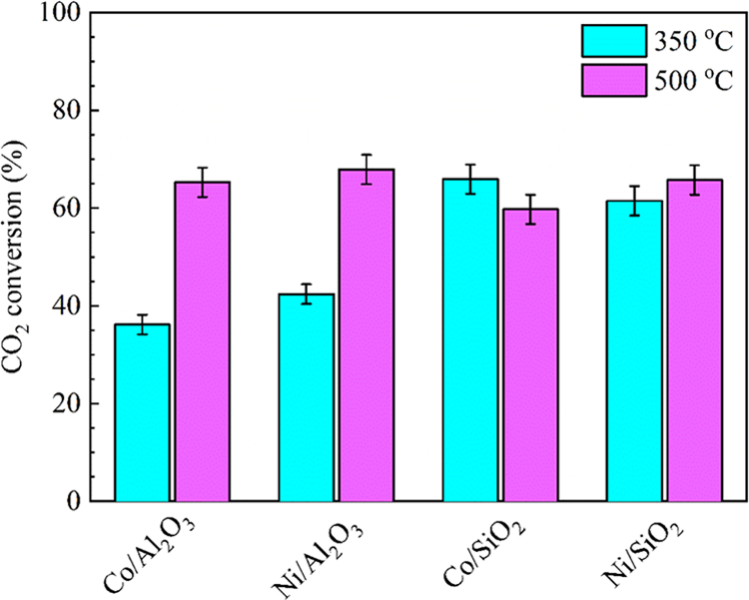
CO_2_ conversions at different reaction temperatures in plasma-catalytic CO_2_ hydrogenation (Ar content: 50%, H_2_/CO_2_ = 4, discharge power: 39 W, total flow rate: 69.2 mL min^–1^).

Figure 7 shows the CO_2_ conversions over M/SiO_2_ and M/Al_2_O_3_ (M = Co, Ni) at varying reaction temperatures using plasma catalysis. The results show that the CO_2_ conversion over the SiO_2_-supported Co and Ni catalysts remained nearly constant when increasing the reaction temperature between 350 and 500 °C. However, the CO_2_ conversion over Al_2_O_3_-supported catalysts increased significantly when increasing the temperature. Among all of the catalysts tested, the highest CO_2_ conversion (67.8%) was obtained using Ni/Al_2_O_3_ at a reaction temperature of 500 °C.

**Figure 8:**
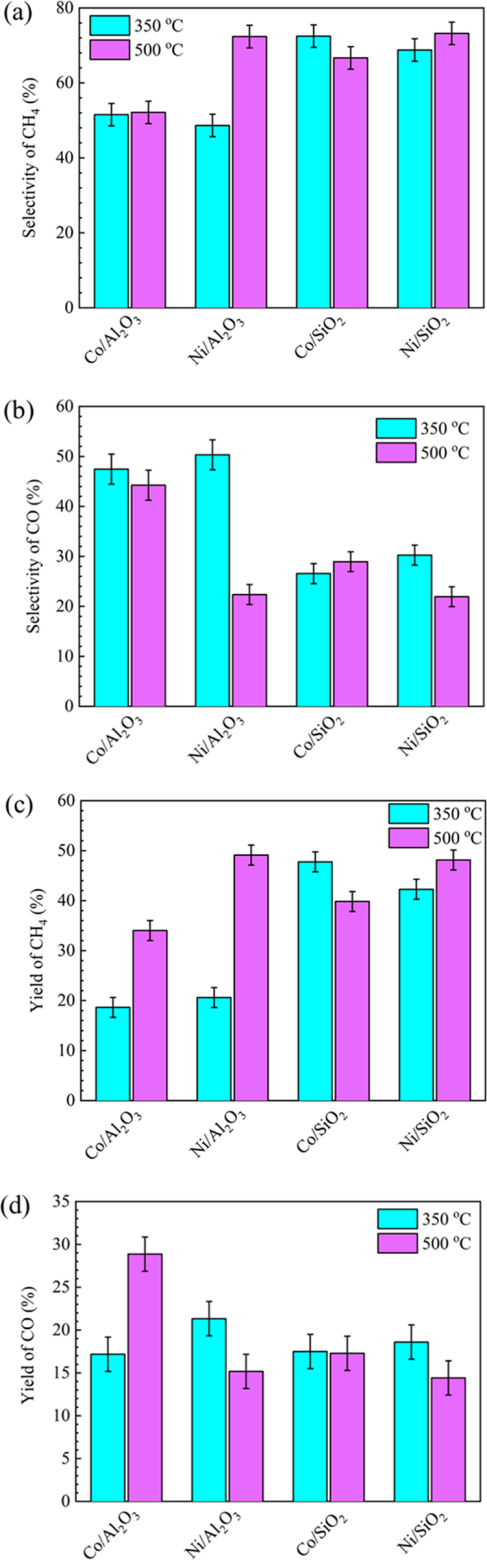
Selectivities (a,b) and yields (c,d) of CH_4_ and CO over M/SiO_2_ and M/Al_2_O_3_ (M = Co, Ni) catalysts at different reaction temperatures in plasma-catalytic hydrogenation processes (Total flow rate: 69.2 mL min^–1^, Ar content: 50%, H_2_/CO_2_ = 4, discharge power: 39 W).

Figure 8 presents the effect of the reaction temperature on the production of the main gases products (CO and CH_4_) using different catalysts in the plasma catalysis system. The selectivity and yields of CH_4_ increased for all of the catalysts except Co/SiO_2_ when the reaction temperature was increased from 350 to 500 °C. Notably, the CH_4_ selectivity increased substantially from 48.6 to 72.4% over Ni/Al_2_O_3_, whereas the CO selectivity dropped from 50.3 to 22.4%. Similarly, the CH_4_ selectivity achieved using the Co/SiO_2_ catalyst dropped from 71 to 67%, while the CO selectivity slightly increased from 26 to 29%.

Table 3 shows the carbon balances of plasma-catalytic CO_2_ hydrogenation using different catalysts at reaction temperatures of 350 and 500 °C. Although the carbon balance slightly decreased as the reaction temperature increased, it remained above 99%. Table 4 presents a summary of the results obtained from various catalysts tested for plasma-assisted CO_2_ methanation in DBD reactors. The maximum levels of CO_2_ conversion and CH_4_ yield obtained in this work were comparable to those in previous studies conducted under similar conditions.

**Table 3:** Carbon Balances of Plasma-Catalytic CO_2_ Hydrogenation Using Different Catalysts at Reaction Temperatures of 350 and 500 °C (Ar Content: 50%, H_2_/CO_2_ = 4, Discharge Power: 39 W, Total Flow Rate: 69.2 mL min^–1^)

	carbon balance (%)	
catalyst	350 °C	500 °C
Co/Al_2_O_3_	99.7	99.4
Ni/Al_2_O_3_	99.6	99.3
Co/SiO_2_	100	99.4
Ni/SiO_2_	100	99.4

**Table 4:** Plasma-Catalytic CO_2_ Hydrogenation Using DBD Reactors

**catalyst**	**H**_**2**_**/CO**_**2**_	**total feed flow (mL min^–1^)**	**P (W)**	**reaction temperature (°C)**	**CO**_**2**_**conversion (%)**	**CH**_**4**_**yield (%)**	**CH**_**4**_**selectivity (%)**	**refs**
15Ni/γ-Al_2_O_3_	4	69	30	150	29	1.15	3.9	(44)
5Co/CeZrO_4_	4	25	N/A	150	70	69.3	99	(45)
6Ru/γ-Al_2_O_3_	3	50	N/A	25	12.8	9.4	73	(50)
10Ni/γ-Al_2_O_3_	4	250	35	170	61	57	93	(43)
Ce–Ni/Cs-USY	4	250	35	170	79	77.4	98	(43)
15Ni/Cs-USY	4	250	35	170	75	72	96	(43)
Ni–Ce,Zr(Mg,Al)O	4	200	16	260	68	56	82.3	(51)
Ni/La-ZrO_2_	1	100	4.9	343	27.7			(52)
Cu/La-ZrO_2_	1	100	4.9	393	27			(52)
Co/SiO_2_	4	69.2	39	350	68	48	71	this work
Ni/Al_2_O_3_	4	69.2	39	500	67.8	49.1	72.4	this work

USY: Ultrastable Y zeolite.

## 4 Conclusions

This work investigated the plasma-catalytic CO_2_ hydrogenation over M/SiO_2_ and M/Al_2_O_3_ (M = Co, Ni) catalysts at different reaction temperatures. The results show that the CO_2_ conversions in plasma-catalytic processes were higher than those in thermal-catalytic processes across all catalysts, indicating a plasma-catalytic synergy. In addition, the catalysts with SiO_2_ support performed significantly better than the Al_2_O_3_-supported metal catalysts in terms of CO_2_ conversion in both plasma-catalytic and thermal-catalytic modes at a reaction temperature of 350 °C. This is likely due to the higher reducibility and larger specific surface areas of the SiO_2_-supported catalysts. The catalytic performance of SiO_2_-supported catalysts was hardly affected by increasing the reaction temperature from 350 to 500 °C. However, the activity of Al_2_O_3_-supported catalysts significantly increased when the reaction temperature was increased to 500 °C. Overall, this study demonstrated that the use of plasma with SiO_2_-supported Co and Ni catalysts could significantly enhance the performance of hydrogenation of CO_2_ to CH_4_ at low temperatures.
